# Compartmentalization of self-representations in female survivors of sexual abuse and assault, with posttraumatic stress disorder (PTSD)

**DOI:** 10.1017/S0033291719000837

**Published:** 2020-04

**Authors:** Georgina Clifford, Caitlin Hitchcock, Tim Dalgleish

**Affiliations:** 1 Medical Research Council Cognition and Brain Sciences Unit, University of Cambridge, 15 Chaucer Road, Cambridge, CB2 7EF, UK; 2 Cambridgeshire and Peterborough NHS Foundation Trust, University of Cambridge, 15 Chaucer Road, Cambridge, CB2 7EF, UK

**Keywords:** Autobiographical memory, compartmentalization, overgeneral, Post-traumatic stress disorder, self-concept

## Abstract

**Background:**

This study examined the structure of the self-concept in a sample of sexual trauma survivors with posttraumatic stress disorder (PTSD) compared to healthy controls using a self-descriptive card-sorting task. We explored whether individuals with PTSD possess a highly affectively-compartmentalized self-structure, whereby positive and negative self-attributes are sectioned off into separate components of self-concept (e.g. self as an employee, lover, mother). We also examined redundancy (i.e. overlap) of positive and negative self-attributes across the different components of self-concept.

**Method:**

Participants generated a set of self-aspects that reflected their own life (e.g. ‘self at work’). They were then asked to describe their self-aspects using list of positive or negative attributes.

**Results:**

Results revealed that, relative to the control group, the PTSD group used a greater proportion of negative attributes and had a more compartmentalized self-structure. However, there were no significant differences between the PTSD and control groups in positive or negative redundancy. Sensitivity analyses demonstrated that the key findings were not accounted for by comorbid diagnosis of depression.

**Conclusion:**

Findings indicated that the self-structure is organized differently in those with PTSD, relative to those with depression or good mental health.

## Introduction

There are profound individual differences in the way we process and organize information related to our self-concept – our experienced sense of self. Theoretical accounts of how the self-concept is structured propose that it comprises multiple ‘self-aspects’ – distinct identities that are represented by organised bodies of both declarative and episodic knowledge (e.g. Cantor and Kihlstrom, 1987; McConnell, 2011). Self-aspects can include roles (e.g. mother, teacher) (e.g. Roberts and Donahue, 1994), social identities (e.g. being a Muslim, a member of the UK Labour party), social relationships (e.g. friend, wife), affective states (e.g. ‘when I'm depressed’), behavioral situations (e.g. ‘when I'm meeting new people’), private and public selves (e.g. Triandis, 1989), and relational and collective identities (e.g. Brewer and Gardner, 1996). Self-aspects are conceptualized as cognitive structures containing sets of specific attributes or beliefs (Showers *et al.*, 2006) that prototypically include significant amounts of affect-laden information (Cantor *et al.*, 1986). It is proposed that different self-aspects will preside over mental experiences in different contexts – what we have previously called the ‘self-in-place’ (Dalgleish and Power, 2004). So, the ‘self with family’ self-concept would preside when an individual is with their family, whereas the ‘depressed self’ would drive self-related experiences when the individual is under the yoke of depressed mood. Under such circumstances, the attributes, beliefs and affect associated with the presiding self-aspect will be more accessible relative to information pertaining to self-aspects that are subordinate in that context.

### Affective compartmentalization

It is proposed that self-concepts can vary in complexity across individuals. Linville (1987) argued that a more complex self-concept is characterized by a greater number of self-aspects and stronger distinctions or boundaries between different self-aspects, in other words, the degree to which the self-concept is compartmentalized. There has been increasing interest in the relationship between the structure of the self-concept and mental health, with a particular focus on how affective self-related information is organized across different self-aspects and how this relates to different degrees of self-concept complexity.

Two aspects of how affective information is organized seem particularly important for mental health. The first is the degree to which affective material is compartmentalized such that positive and negative self-attributes are segregated into separate self-aspects (Showers, 2002). For an individual with a high degree of affective compartmentalization, any given self-aspect (e.g. ‘self at work’, ‘self with friends’) will be dominated by either positive (e.g. happy, confident) or negative (e.g. worried, hopeless) self-attributes, as opposed to a self-aspect being represented by a balance of positive and negative attributes (e.g. happy, worried). For example, an affectively compartmentalized person may have a positive self-aspect category (e.g. ‘self with close friends’), which contains predominantly positive conceptualizations about that instantiation of the self (e.g. confident, optimistic, happy and organized). As long as such positively valenced self-aspects are salient, these primarily positive self-beliefs will populate conscious awareness with consequent implications for affect and well-being. Conversely, when negatively valenced self-aspects are salient, the phenomenology of highly compartmentalized individuals would be dominated by negative self-beliefs. For example, for a highly compartmentalized person with a negative self-aspect category (e.g. ‘self at work’), it is proposed that highly accessible negative self-related beliefs (e.g. worried, hopeless, uncomfortable and insecure) will dominate mental life when at work.

Several authors have discussed how high levels of affective compartmentalization may arise out of stressful or traumatic formative experiences as a means of ‘ring fencing’ off toxic self-related material from more positive self-aspects (Linville, 1987; Morgan and Janoff-Bulman, 1994; Showers *et al.*, 2006; cf. also Steinberg *et al.*, 2003). Such affective compartmentalization can be viewed as both a protective strategy and as a vulnerability factor. When positively-valenced compartmentalized self-aspects preside over mental life, difficult or toxic self-related information is kept psychologically at bay, promoting experiential well-being (Linville, 1987). However, to the extent that the individual is vulnerable to the self-in-place (Dalgleish and Power, 2004) being occupied by a predominantly negative self-aspect, characterized by self-attributes grounded in experiences of significant unresolved stress or trauma, then such compartmentalization represents a risk factor for mental distress or ill health.

The counterpart to this compartmentalized structure is the notion of an integrated self-concept characterized by a mixture of positive and negative self-attributes *within* most or all self-aspects (Showers, 2002). An individual with a highly-integrated self-concept may also endorse the self-aspect – ‘self at work,’ – but in this case, this self-aspect would contain a balance of both positive and negative self-content. Although such individuals may not inhabit self-aspects with unremittingly positive content they are also less susceptible to the toxic override potential of highly negative self-aspects and consequently have reduced mental health vulnerability. This has been corroborated across numerous studies linking self-concept integration with mental health and well-being (e.g. Showers, 1992; Showers and Kling, 1996; Rhodewalt *et al.*, 1998; Showers *et al.*, 1998).

The first aim of the present study was to extend this work on self-structure and mental health to look at individuals who had experienced significant trauma – in this case sexual abuse and/or assault – and who are suffering as a consequence from Post-Traumatic Stress Disorder (PTSD). PTSD is characterized by negative beliefs about the self being broken or damaged in some way by the trauma. For example, one criterion in the DSM-5 [American Psychiatric Association (APA), 2013] diagnosis for PTSD is *‘Persistent and exaggerated negative beliefs or expectations about oneself, others, or the world* (*e.g. “I am bad,” “No one can be trusted,”*)*’*. Sufferers of PTSD also invariably possess a rich repertoire of psychological and behavioral strategies to protect against potentially toxic information about their trauma and its implication or consequences for the self. Many of these comprise the DSM-5 avoidance symptoms of PTSD (avoiding trauma reminders, attempts to never think about or talk about the trauma, social withdrawal, loss of interest in activities, emotional numbing and psychogenic amnesia) (APA, 2013). Others involve associated phenomena such as dissociation (including depersonalization and derealization), suppression and repression, which sufferers of PTSD often use to inhibit the reliving symptoms and overwhelming emotions associated with the trauma (Holmes *et al*., 2005).

Based on these aspects of the PTSD phenotype and on the theoretical literature outlined above, our first hypothesis was that individuals with PTSD following an experience or experiences of interpersonal trauma such as sexual abuse or assault would possess a highly affectively-compartmentalized self-structure relative to individuals who have not experienced sexual trauma and do not suffer PTSD.

### Affective redundancy

The second organizational principle relating the structure of the self-concept to mental health is the extent to which affective self-related knowledge shows overlap or *redundancy across* (Linville, 1987) different self-concepts. For example, the self may be represented as ‘worthless’ across multiple self-aspects such as ‘self as a friend’, ‘self at work’, ‘self as a spouse’ (e.g. Dozois and Dobson, 2001*a*, 2001*b*; Linville, 1985; Dalgleish and Power, 2004). In such circumstances, potentially toxic negative information is not effectively confined or compartmentalized into discrete self-aspects but pervades the individual's entire sense of self, potentially contaminating even the most positive self-aspects. In contrast, high redundancy of positive information would reflect a stable positive sense of self, with beneficial consequences for mental health and well-being. Unsurprisingly, perhaps, research on the structure of the self-concept in those with clinical depression reveals greater overall negativity, greater redundancy of negative attributes across self-aspects, reduced positive redundancy, and stronger affective compartmentalization than is the case for those who have never suffered from depression. (e.g. Showers and Zeigler-Hill, 2007; Dalgleish *et al*., 2011).

The second aim of the present study was to examine redundancy of positive and negative self-attributes across the self-concept in trauma-exposed individuals with PTSD and the healthy control participants. We had a clear hypothesis regarding positive redundancy, predicting that it would be reduced in the individuals with PTSD, reflecting the absence of a stable positive sense of self. We had no clear hypothesis regarding negative redundancy. It is plausible that the repertoire of inhibitory strategies that characterizes PTSD would serve to corral negative self-related information into a small number of negatively-laden self-aspects with little ‘spillover’ or redundancy with the rest of the self-concept. In contrast, it is also plausible that the content of any negative self-attributes that had their origins in the person's experience of trauma would generalize to pervasive negative self-representations that populated the entire self-concept, akin to the pervasive negativity observed in depression (Dalgleish *et al*., 2011).

### Self-descriptive card sort

To examine the structure of self-concept we used a self-descriptive card-sorting task (Showers, 1992).In this card sorting procedure, participants are first asked to generate a set of self-aspects that reflect their own life (e.g. ‘self at work’, ‘self when angry’). There can be as many, or as few, self-aspects as seem relevant to a given individual. Participants are then presented with a set of 48 cards, each containing a trait word or phrase which is either positive or negative in valence.

The participants are asked to sort the cards into one, many or none of the self-generated self-aspects (Linville, 1985, 1987).^1^ Within this procedure the degree of negativity (Showers, 1992) is the overall proportion of cards selected that are negative in valence across all self-aspects, including repetitions. Redundancy or overlap is the extent to which the same cards are used across multiple self-aspects (Dozois and Dobson, 2001*a*, 2001*b*). Finally, affective compartmentalization is the extent to which positive and negative cards are allocated to distinct self-aspects such that some self-aspects are predominantly positive, while others are predominantly negative (Showers, 1992).

### The current study

The principal focus of the present study was to examine degree of negativity, positive and negative redundancy, and compartmentalization of valenced information across the self-generated self-aspects of an individual's self-concept, as revealed by the card sort procedure, in a sample of participants with current PTSD following significant interpersonal trauma, relative to healthy controls who had not experienced such trauma. Although compartmentalisation and other effects of the self-structure may occur following the experience of any trauma, we anticipated that these effects would be particularly likely following sexual trauma, due to the violation of both physical and self-integrity, and prevalence of more intense avoidance strategies such as dissociation, and documented effects on the self-concept (e.g. Finkelhor and Brown, 1985; McAlpine and Shanks, 2010).

In sum, we predicted that the PTSD group would identify the most stressful or traumatic event in their lives as more centrally defining in terms of how they see themselves, relative to the controls, as measured by the Centrality of Events Scale (Berntsen and Rubin, 2006, 2007) – a self-report inventory assessing how identified events have come to define your personal identity. In terms of the card-sorting task, we hypothesized that all participants would generate multiple self-aspects but that the PTSD sample would display greater negativity across the self-concept as well as greater compartmentalization between positive and negative components of the self-concept, across their different self-aspects. We also predicted that redundancy of positive information across the different self-aspects would be reduced in those with PTSD relative to the controls, but we had no clear directional hypotheses regarding negative redundancy.

## Method

### Participants

Power analysis was completed in G Power. We used the effect size (*d* = 1.02) for the difference between healthy and depressed samples in card-sort metrics observed by Dalgleish *et al*. (2011), as we anticipated that the size of the effect may be similar for the difference between PTSD and healthy samples. The calculation indicated that 22 participants per group would provide 90% power (two-tailed, *α* = 0.05) to detect a true effect. We therefore aimed to recruit 22 participants into each group.

Two groups of female participants were included in the study. Participants who had developed PTSD following sexual trauma were allocated to a PTSD group. Current diagnosis of PTSD was determined according to the DSM-IV (*n* = 23). Fifteen of these participants were recruited from the Haven; A Sexual Assault Referral Centre (SARC) in Paddington. Following the completion of an assessment for counseling or psychological therapy at the Haven, potential participants were given an information sheet for the study, and invited to contact the researchers if they would like to take part. Eight participants were recruited from the MRC Cognition and Brain Sciences Unit Clinical Volunteer Panel – a database of some 400 community volunteers with a history of significant mental health problems who have agreed to help with psychological research. Volunteers are recruited to the panel via advertisements in local newspapers and through local clinics.

PTSD diagnosis and history and other Axis I and II psychiatric comorbidity according to the DSM-IV were determined using the Structured Clinical Interview for the *DSM-IV* Axis I Disorders – Clinician Version (SCID, Version 2.0; First *et al.*, 1996) and the Structured Clinical Interview for DSM-IV-TR Axis II Personality Disorders (Borderline, Avoidant and Dependant) (First *et al.*, 1997), administered by trained interviewers, under the supervision of a Clinical Psychologist.

The female participants with no experience of sexual abuse/assault and without PTSD (which may have occurred from other events such as motor vehicle accidents) as determined by the SCID (the control group; *n* = 22), were recruited from the MRC Cognition and Brain Sciences Unit Non-Clinical Volunteer Panel – a database of some 2000 community volunteers who have agreed to help with psychological research. Volunteers are recruited to the panel via advertisements in local newspapers.

To be eligible for the study, participants had to be fluent in English and over 18 years of age. Exclusion criteria comprised a diagnosis of substance dependence, organic brain injury and a history of psychosis. No participants were excluded based on this criteria.

### Materials and measures

#### Self-structure card-sorting task

The card-sorting task was adapted from Showers (1992; Showers and Kling, 1996; Showers and Kevlyn, 1999), although the original task was proposed by Zajonc (1960) and subsequently adapted by Linville (1985, 1987). First, participants were given a description of how we define ‘self-aspects.’ Participants were then asked to identify and describe each of their different ‘self-aspects’. They were told that they were free to come up with as many different self-aspects as they felt were appropriate. Participants were given a blank table and asked to record their self-aspects at the top of a column.

Participants were given a deck of 48 cards (listed in online Supplementary Appendix A), shuffled anew for each participant. Each card contained an adjective or phrase that might be used to describe a self-aspect. Participants were asked to record which of the cards fell under each self-aspect. Any card can be used repeatedly if it is relevant to more than one self-aspect, or not at all if it is deemed irrelevant to the self. The adjectives chosen were modified from Dalgleish *et al*.’s (2011) study to be more specifically trauma-related. For example, *‘feeling contaminated’, ‘feeling broken’* and *‘feeling dirty.’* The adjectives/phrases were either positive or negative in valence (24 of each; see online Supplementary Appendix A). Prior to the study, we had the adjectives/phrases rated (*n* = 15 raters) for valence on 15-point Likert scales anchored at 1 (*strongly positive*), 7 (*weakly positive*), 8 (*neutral*), 9 (*weakly negative*), 15 (*strongly negative*). The positive set of adjectives had a mean rating of 2.59 (s.d. = 0.81), whereas the negative set of adjectives had a mean rating of 13.61 (s.d. = 1.09). A paired samples *t* test showed that the two sets of cards did not differ significantly in intensity (distance from the neutral score of 8; *t* < 1).

We also asked participants to think about their ‘core self’ – the parts of their self concept that they felt were always almost experientially present and that underlay their experience of their different self-concepts. Participants were provided with a definition and then asked to take the 48 cards and then select those which they felt described their ‘core-self.’. We hypothesized that the core self in our sample with PTSD would be more negatively laden than in our control group.

### Self-structure metrics

#### Proportion of negative items

This is the number of negative words or phrases, including repetitions, appearing in the card sort, divided by the total number of words or phrases used. It is a measure of the overall negativity of the sort (Showers, 1992).

#### Compartmentalization (Showers, 1992)

The measure of compartmentalization is a phi (*φ*) coefficient based on a χ^2^ statistic (Everitt, 1977). It compares the frequencies of positive and negative cards in each self-aspect of the card sort to those that would be expected, given the proportion of negative items for the sort as a whole. A frequency table is constructed that contains as many columns as there are self-aspects in the individual's card sort and one row each for number of positive cards and number of negative cards. The observed frequencies for each cell are generated from the whole card sort. The expected frequencies are generated as follows: If the card sort contained, for example, 40% negative cards overall and the first self-aspect contained 20 cards, then the expected frequencies for that aspect would be 8 (40%) negative cards and 12 (60%) positive cards. A χ^2^ statistic is then computed using these expected and observed frequencies. This is then normalized by dividing by the number of cards in the sort (*N*) as follows:
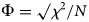
where, *φ* can range from 0 to 1 (0 represents a perfectly random sort, and 1 represents a perfectly compartmentalized sort).

#### Redundancy

Redundancy (Dozois and Dobson, 2001*a*, 2001*b*) was computed separately for positive and negative attributes, with each redundancy score representing the degree of card repetitions across self-aspects, controlling for both the number of self-aspects in a given card sort and the number of cards used. The following formula generated the redundancy rates:
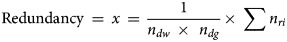
where (using the example of negative redundancy) *n*_*dw*_ equals the number of distinct negative words used in an individual's card sort, *n*_*dg*_ equals the number of self-aspects generated, and ∑*n*_*ri*_ equals the sum of repetitions of each negative card up to the maximum of 23 cards.^2^

### Screening and questionnaire measures

#### SCID-I for mood disorders; anxiety and other disorders

Axis I diagnoses according to the *Diagnostic and Statistical Manual of Mental Disorders* (4^th^ ed.; *DSM-IV*; American Psychiatric Association, 1994) were determined by having participants complete the Structured Clinical Interview for the *DSM-IV* Axis I Disorders – Clinician Version (SCID, Version 2.0; First *et al.*, 1996) under the supervision of a Clinical Psychologist. The reliability and validity of the SCID-I for DSM-IV has been reported in several published studies (e.g. Zanarini *et al*., 2000; Lobbestael, *et al*., 2011).

#### SCID-II (borderline, avoidant and dependent personality disorder sub-sections)

Diagnoses of Borderline, Avoidant and Dependent Personality Disorder were determined by having participants complete the Structured Clinical Interview for DSM-IV Axis II disorders (SCID-II; First *et al.*, 1997). Excellent inter-rater reliability has been found on the SCID-II (range 0.77 to 0.94). The intraclass correlation coefficient (ICC) trait scores of all personality disorders were excellent, with the exception of the schizotypal, histrionic, narcissistic and the A criteria of antisocial personality disorders which displayed fair inter-rater agreement (e.g. Lobbestael, *et al*., 2011).

#### Beck depression inventory (BDI-I; Beck *et al.*, 1961)

The Beck Depression Inventory (BDI; Beck *et al.*, 1961) is a widely-used and well validated measure of depressive symptoms over the previous week.^3^ The BDI demonstrates high internal consistency, with alpha coefficients of 0.86 and 0.81 for psychiatric and non-psychiatric populations respectively (Beck *et al*., 1988). Internal consistency was high in the current sample (*α* = 0.96).

#### Centrality of events scale (CES-Negative; Berntsen and Rubin, 2006, 2007)

The CES-Negative (Berntsen and Rubin, 2006, 2007) measures the extent to which a negative or traumatic memory forms a central component of personal identity, a turning point in the life story, and a reference point for everyday inferences. We used the full version, which consists of 20 items rated on 5-point scales (1 = totally disagree to 5 = totally agree) in relation to the most stressful or traumatic event in the person's life. The CES-negative is positively correlated with severity of PTSD symptoms, and this relationship remains significant when controlling for measures of anxiety, depression, dissociation, and self-consciousness (Berntsen and Rubin, 2006; 2007). Internal consistency for the CES was high in the current sample (*α* = 0.98).

### Procedure

Ethics approval was obtained from the NHS National Research Ethics Service (reference 11/H0305/1). Participants completed the experimental session individually and face-to-face with the experimenter, in a quiet, private testing room, with only the experimenter present. Following provision of written informed consent, participants completed the SCID and several self-report questionnaire measures of mood and PTSD symptoms. In a separate session, approximately a week later, they completed the self-structure card sort. Participants were instructed to take their time in completing the tasks and were repeatedly reminded that they could stop at any time if they felt distressed. However, all participants finished their testing sessions and reported experiencing manageable levels of distress throughout the session, in response to experimenter queries.

## Results

### Participant characteristics

According to the SCID, of the 23 participants in the PTSD group, nine also met the criteria for a current episode of Major Depressive Disorder (MDD), 19 met the criteria for a past episode of MDD, one for current panic disorder (secondary to PTSD), four for current Borderline Personality Disorder (BPD) and two for current Avoidant Personality Disorder. Due to the complex nature of our participants' trauma histories and their experience of repeated events, we were not able to obtain an accurate estimation of time since the last traumatic event. During the SCID, the median number of experienced traumatic events was ‘too many to count’. In the control group, one participant met the criteria for a current episode of MDD and five met the criteria for a past episode of MDD. The participant in the control group who met criteria for a current episode of MDD was excluded from the analyses.

Descriptive group data are presented in Table 1. The groups did not differ significantly in age, *t*_(42)_ = 1.41, *p* = 0.17, *d* = 0.44; 95% CIs (−0.03 to 2.63), nor in education level, *t*_(42)_ = 1.75, *p* = 0.09, *d* = 0.54; 95% CIs (−0.17 to 2.77), although there was tendency for the PTSD group to report fewer years in education. There were the expected differences in BDI scores between the PTSD and Control groups (BDI: *t*_(24.01)_ = 7.35, *p* < 0.001, *d* = 3.00; 95% CIs (−2.16 to 4.76); and support for our first hypothesis that the PTSD group would identify the negative events they had experienced as more self-defining on the CES: *t*_(37.63)_ = 6.79, *p* < 0.001), *d* = 2.21; 95% CIs (−1.67 to 4.27).

**Table 1.** Mean (standard deviation) for sample characteristics

Category	PTSD group (*n* = 23)	Control group (*n* = 21)
Years in education	14.87 (2.32)	16.05 (2.13)
Age in years	35.87 (14.03)	30.19 (12.54)
Beck Depression Inventory (BDI-I)	24.77 (12.83)	3.95 (3.37)
Centrality of Events (CES) Total score	80.00 (15.49)	42.76 (20.07)

### Self-structure

All participants were able to come up with multiple self-aspects (range 2–15). Examples of self-aspects were ‘self at work’, ‘self with close friends’, self with men’, and ‘self at home.’

**Fig. 1.**
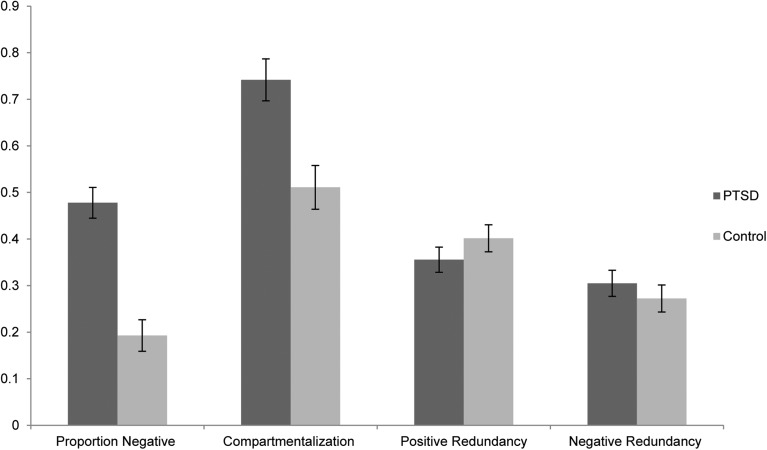
Mean (s.e.) performance (*y*-axis) for the PTSD and control groups for Proportion of Negative cards used, Positive and Negative Redundancy, and Compartmentalization across their multiple self-aspects.

The self-structure data are presented in Table 2 and Fig. 1. The groups were not significantly different on the total number of self-aspects generated, *t*_(42)_ = 1.02, *p* = 0.31, *d* = 0.31; 95% CIs (0.19–2.41), nor the total number of cards used, *t*_(42)_ = 1.54, *p* = 0.13, *d* = 0.48; 95% CIs (−0.09 to 2.69), although participants in the PTSD group used numerically more cards on average. This suggests comparable engagement in the task across groups and indicates that any group differences on the structure metrics considered below, which nevertheless control for overall numbers of self-aspects and cards used, were not likely to be a function of the number of self-aspects generated.

**Table 2.** Mean (and standard deviation) numbers of self-aspects and cards used, by group

Variable	PTSD group (*n* = 23)	Control group (*n* = 21)
Number of self-aspects (range 2–15)	6.70 (2.98)	5.90 (2.02)
Cards used in sort (range 10–229)	75.04 (56.69)	53.90 (28.19)
Cards per self aspect (range 1–36)	10.59 (6.11)	9.27 (4.01)

There were broad ranges of scores across both groups on the four self-structure metrics (maximum possible range 0 to 1) suggesting that across-group floor and ceiling effects were not at work in the data: Proportion of Negative Cards: 0.02–0.92; Negative Redundancy: 0–0.67; Positive Redundancy: 0.18–0.74; and Compartmentalization: 0 to 1. In illustration of the raw data, online Supplementary Appendix B shows two examples of actual card sorts from participants in the control group illustrating relatively high and low levels of Affective Compartmentalization. Of particular note is how, in the integrated card sort example illustrating relatively low Compartmentalization (online Supplementary Appendix B1), several of the self-aspects contain positive and negative descriptors that are diametrically opposite in meaning.

The first analysis assessed whether, overall, self-structure differed across the two groups. To that end we conducted a Multivariate Analysis of Variance (MANOVA) with the four self-structure metrics as the dependent variables.

There was a statistically significant difference in the self-structure components based on group, Wilk's *Λ* = 0.41, *F*_(4, 39)_ = 13.85, *p* = 0.00, *η*_*p*_^*2*^ = 0.59.

Follow-up Univariate ANOVAs showed a significantly greater Proportion of Negative cards [*F*_(1, 42)_ = 36.14, *p* = 0.00, *η*_*p*_^*2*^ = 0.46; 90% CIs (0.26, 0.59)] and significantly greater Compartmentalization [*F*_(1, 42)_ = 12.50, *p* = 0.001, *η*_*p*_^*2*^ = 0.23; 90% CIs (0.07, 0.39)] for the PTSD group. There were no significant differences between groups for Positive or Negative Redundancy, *F*s < 1

### Sensitivity analysis examining the effects of comorbid depression

It is possible that that experience of comorbid clinical depression may have impacted our results. To minimise any variance attributable to comorbid depression, we set aside the nine participants with a comorbid diagnosis of MDD from the PTSD group in order to perform a sensitivity analysis addressing this possibility. When we reran the analyses with the remaining PTSD sample, the self-structure metrics were similar to the whole sample (*n* = 14; see Table 3). Specifically, a MANOVA with the four self-structure metrics again revealed a statistically significant difference in the self-structure components based on group, Wilk's *Λ* = 0.46, *F*_(4, 30)_ = 8.99, *p* = 0.00, *η*_*p*_^*2*^ = 0.55. Univariate ANOVAs again showed a significantly greater proportion of negative cards used, *F*_(1, 33)_ = 26.28, *p* = 0.00, *η*_*p*_^*2*^ = 0.44; 90% CIs (0.22, 0.59) and significantly greater compartmentalization [*F*_(1, 33)_ = 14.42, *p* = 0.002, *η*_*p*_^*2*^ = 0.30; 90% CIs (0.10, 0.47)], for the PTSD group. There was again no significant difference between groups on positive or negative redundancy, *Fs* *<* *1*.

**Table 3.** Means and standard deviations of scores on the self-structure metrics for the participants without co-morbid MDD in the PTSD group and the control group

Self-structure metric	PTSD group without MDD (*n* = 14) *Mean* (s.d.)	Control group (*n* = 21) *Mean* (s.d.)	Effect size (*η*_*p*_^*2*^)
Proportion of negative cards	0.42 (0.13)	0.19 (0.13)	0.44
Negative redundancy	0.31 (0.11)	0.27 (0.16)	0.02
Positive redundancy	0.39 (0.15)	0.40 (0.13)	0.003
Compartmentalization	0.79 (0.22)	0.51 (0.22)	0.30

### Core self data

The core-self data are presented in Table 4. The groups were not significantly different on the total number of cards used, *t*_(42)_ = 1.05, *p* = 0.30, *d* = 0.32; 95% CIs (0.17–2.43). As anticipated, the PTSD group used a significantly greater proportion of negative cards to describe their core self, *t*_(34.38)_ = 3.32, *p* = 0.002, *d* = 1.13; 95% CIs (−0.83 to 3.43).

**Table 4.** Mean (and standard deviation) total number of cards and proportion of negative words across groups

Variable	PTSD group (*n* = 23)	Control group (*n* = 21)
Core Self – total number of cards	9.83 (7.03)	7.95 (4.33)
Core Self – proportion of Negative Words	0.38 (0.31)	0.14 (0.16)

## Discussion

The aim of the present study was to examine the structure of self-concept in a sample of sexual trauma survivors with PTSD compared to healthy controls using a self-descriptive card-sorting task (Showers, 1992). Consistent with our predictions, across self-aspects, the PTSD group used a greater proportion of negative cards and had a more compartmentalized self-structure than the control group. However, in contrast to our predictions, there were no significant differences between the PTSD and control group in positive redundancy. We also found no group differences for negative redundancy, where we had no clear predictions. We also demonstrated, unsurprisingly, that those with PTSD characterized their ‘core self’ as more negative relative to the healthy control group. The pattern of findings on the card-sorting task was not simply a function of different numbers of self-aspects or number of cards used across the group, because there were no significant differences on these variables. To account for the previously established effects of depression (Dalgleish *et al*., 2011) on self-structure, we set aside the participants with a comorbid diagnosis of MDD from the PTSD group and the resultant sensitivity analyses showed that the key findings were unchanged, indicating that the results are not accounted for by depression comorbidity.

As noted in the Introduction, a number of authors have suggested that the higher levels of affective compartmentalization may arise out of stressful or traumatic experiences as a way of ‘ring fencing’ off what tends to be highly distressing negative self-related material from more positive self-aspects (Linville, 1987; Morgan and Janoff-Bulman, 1994; Showers *et al.*, 2006; cf. also Steinberg *et al.*, 2003). So, for an individual with PTSD following a sexual trauma, a particular self-aspect, such as ‘self with men’, which encompassed feelings of shame, hopelessness and insecurity might be compartmentalized or split off from other self-aspects, such as ‘self with close friends,’ which were associated with more positive affect.

The fact that we found no support for a difference in negative redundancy between our groups suggests that in our sample with PTSD, distressing or toxic information relating to past traumatic experiences is compartmentalized across the self-structure, rather than pervading the individual's entire sense of self. In this way, negative material is prevented from contaminating the other, more positive self-aspects. This finding contrasts with previous research in individuals with clinical depression who were found to demonstrate greater overall negativity, greater redundancy of negative attributes across self-aspects, reduced positive redundancy, *and* stronger affective compartmentalization than those who had never suffered from depression. (e.g. Showers and Zeigler-Hill, 2007; Dalgleish *et al*., 2011). This suggests that the self-structure is organized differently in those with PTSD, relative to depression.

Similarly, based on these earlier depression findings, we had predicted that positive redundancy would be reduced in those with PTSD relative to controls, reflecting a reduced stable positive sense of self. However, this was not the case. Our results suggest that, although the overall positivity across the self-structure is lower in those with PTSD, as one would expect given the severe and distressing nature of the disorder, and the positive information is more compartmentalized, the positive *content* that is represented is as consistent across the self-structure as it is healthy participants. Again, this suggests clear differences between the self-structure of those with PTSD compared to those with depression. Without a control group with a complex trauma history but no mental health issues, it is difficult to determine whether this unique self-structure results from the experience of PTSD *per se*, or is due to the experience of multiple interpersonal traumas. Either way, current findings do indicate that distortions in self-structure observed in those with a sexual trauma history is distinct from patterns of self-structure in other mental health disorders.

From our results, it appears that compartmentalization may function to protect positive self-concept, as the consistency of positive content was preserved in our PTSD sample. However, longitudinal research is necessary to evaluate whether compartmentalization of the self-structure is a protective or vulnerability factor for symptom development. In turn, such research may demonstrate the clinical utility of actively encouraging trauma-exposed individuals to compartmentalize *v.* integrate different aspects of the self.

The study has some potential limitations. Although we did ask participants to generate their own self-aspects, we did not ask them to generate their own descriptive words to assign to the cards used in the sorting task. This was because we wanted to ensure that there were comparable numbers of positive and negative cards to select from and also to ensure that the intensity of descriptors was comparable across participants so that we could draw conclusions about the structure of the self-concept as opposed to the language used to describe it. Future studies could ask participants to provide their own adjectives to describe each self-aspect, that could then be rated in terms of valence and coded using metrics similar to those employed here (Rubin and Bernsten, 2003).

The second issue is that our clinical group and control group differed in two key ways. The clinical group consisted of a sample of individuals who experienced sexual abuse and/or assault and who, as a consequence, had developed PTSD. Our controls comprised individuals who did not report traumas of this nature and who did not meet criteria for PTSD. This means that it is not possible to disentangle whether it is the development of PTSD rather than the trauma history, *per se*, that can account for differences in negative material and compartmentalization. The reason for this is that it is very difficult to find individuals with this kind of trauma history, at the level of severity of our sample, who are without any mental health problems and so any trauma-matched control group would likely present with significant symptoms of PTSD (alongside diagnoses of other disorders) even though they might not meet criteria for a full diagnosis. Future studies could examine the replicability of the effects with survivors of more discrete or less severe trauma to seek to disentangle the experience of trauma from the presence of PTSD. Such studies would also speak to the generalizability of the effects from severe interpersonal trauma to other trauma categories.

A third issue is that the sample sizes for the two groups were modest as is often the case for hard-to-recruit clinical samples. However, there is no suggestion in the pattern and magnitude of the results that lack of statistical power is responsible for any of the findings. The samples were also all female. It is now important to replicate the findings with larger samples including individuals with PTSD who have experienced different traumas and who are male. And finally, although we draw conclusions about the self-structure in PTSD relative to depression based on comparisons with the previous literature, we have not directly compared a PTSD (with no comorbid depression) group to a clinically depressed (with no comorbid PTSD) group.

In summary, the present study used an established card-sorting task to examine degree of negativity, positive and negative redundancy, and compartmentalization of valenced information across the self-generated self-aspects of an individual's self-concept in a female sample of individuals with PTSD relative to healthy controls. The data revealed a greater proportion of negative cards and a more compartmentalized self-structure in individuals with PTSD, compared to a non-clinical control group, but provided no support for differences in positive or negative redundancy. This is consistent with literature proposing that high levels of affective compartmentalization may arise out of stressful or traumatic experiences as a way of ‘ring fencing’ off negative self-related material from the more positive self-aspects. These data fit with our understanding of PTSD and the mechanisms involved, such as avoidance and dissociation, that are used to inhibit the negative impact of past traumatic experiences.

## Notes

1 For example, a card may contain the adjective ‘confident’ and, during the card sort, the participant would decide how many, if any, of his/her self-aspects could be described in this way and allocate that card accordingly.

2 The prototypical work on self-structure by Linville (1985, 1987) used a metric of ‘self-complexity,’ which is a combination of overlap or redundancy across self-aspects and the overall numbers of self-aspects generated. However, self-complexity is highly correlated with numbers of different cards used in each card sort (Woolfolk *et al.*, 1995), which militates against its use. We did not anticipate group differences in numbers of self-aspects generated in the present study and we were careful to match our groups on mean age. For these reasons among others (Dozois and Dobson, 2001*a*, 2001*b*; Rafaeli-Mor *et al.*, 1999), we did not use the self-complexity metric in the present study.

3 The original BDI was preferred here over updated versions for legacy reasons because the Cognition and Brain Sciences Unit Volunteer Panel has an extensive historical database of original BDI scores that can be used when recruiting for studies.

## Supplementary material

For supplementary material accompanying this paper visit https://doi.org/10.1017/S0033291719000837. click here to view supplementary material
