# Supplementary material for: Compartmentalization of self-representations in female survivors of sexual abuse and assault, with posttraumatic stress disorder (PTSD)
Source: Psychol Med. 2019 Apr 23;50(6):956–63. doi: 10.1017/S0033291719000837 (PMC7191781; doi:10.1017/S0033291719000837)
Supplement: Supplementary file 1 [file S0033291719000837sup001.doc]

**Appendix A**

Positive and negative words/phrases used in the Card Sorts

| Positive | Negative |
| --- | --- |
| Happy  Satisfying  Overjoyed  Fulfilling  Successful  Feeling Loved  Confident  Creative  Feeling Needed  Passionate  Feeling Nurtured  Joyful  Wisdom  Accomplished  Important  Feeling Together  Exciting  Complete  Relaxed  Feeling Courageous  In Control  Organised  Stable  Feeling Pure | Naïve  Incomplete  Confused  Boring  Apathetic  Moody  Regretful  Feeling Contaminated  Stressful  Out of Control  Unsuccessful  Feeling Broken  Insignificant  Insecure  Ashamed  Feeling Rejected  Unfulfilling  Gloomy  Feeling Unwanted  Lonely  Depressing  Feeling Unloved  Hopeless  Failure |
|  |  |

*(Appendices continue)*

**Appendix B**

**Sample self-structure card sorts for two Control group participants**

Table B1

Card Sort for a control group participant with a predominantly positive self-structure and a relatively integrated mix of positive and negative words (Φ= 0.28)

| Friends | Relationship | Colleagues | Alone | Travelling | Home |
| --- | --- | --- | --- | --- | --- |
| *Creative*  *Happy*  *Confident*  *Important*  *Loved*  *Fulfilling*  *Joyful*  *Needed*  *Together*  *Complete*  *In control*  *Stable*  *Relaxed* | *Exciting*  *Nurtured*  *Relaxed*  **Confused**  *Stable*  *Satisfying*  *Together*  *Passionate*  *Needed*  **Stressful**  *Joyful*  *Fulfilling*  *Loved*  *Important*  *Happy* | *Organised*  *Wisdom*  *Satisfying*  *In control*  **Out of control**  *Courageous*  **Naive** | *Exciting*  *Courageous*  **Naive**  *Relaxed*  *Stable*  **Insecure**  *Accomplished*  *In control*  **Out of control**  *Together*  *Needed*  **Lonely**  *Wisdom*  **Stressful**  *Loved*  *Important*  *Creative*  **Moody** | *Stable*  *Confident*  *Importan*  *Loved*  *Fulfilling*  *Joyful*  **Confused**  *Wisdom*  **Stressful**  *Together*  **Lonely**  *Complete*  *Satisfying*  **Out of control**  *In control*  *Relaxed*  **Naive**  *Accomplished*  *Courageous*  *Exciting* | *Couraegous*  *Nurtured*  *Relaxed*  *Stable*  *In control*  *Satisfying*  *Together*  *Needed*  *Pure*  *Joyful*  **Moody**  *Loved*  *Important*  **Boring**  *Happy* |

*Note:* Self-aspect titles have been adapted slightly for the purpose of anonymity, though they remain faithful to the content. Negative words are in bold, and positive words are in italics.

*(Appendices continue)*

Table B2

Card Sort for a control participant with a more compartmentalized structure (Φ= 0.90)

| With parents | With my brother | With friends | At school | With boyfriend | When unwell | When alone |
| --- | --- | --- | --- | --- | --- | --- |
| *Organised*  *Accomplished*  *Feeling Loved*  *Nurtured*  **Stressful**  **Boring**  *Joyful*  *Stable*  **Out of Control**  *Confident*  *Important*  *In control* | *Organised*  **Stressful**  **Insecure**  **Boring**  **Regretful**  **Out of Control** | *Organised*  *Happy*  *Feeling Loved*  *Joyful*  *Courageous*  *Relaxed*  *Exciting*  *Creative*  *Important*  *Feeling needed*  *Confident*  *Satisfying*  *Passionate*  *Feeling Together*  *In control* | *Organised*  *Accomplished*  **Insignificant**  **Stressful**  **Confused**  **Insecure**  **Lonely**  **Unsuccessful**  **Out of Control**  **Apathetic**  **Failure** | *Happy*  *Feeling Loved*  *Nurtured*  *Feeling Together*  *Stable*  *Feeling Needed*  *Joyful*  **Ashamed**  *Relaxed*  *Creative*  *Satisfying*  *Important*  *Confident*  *Passionate*  *Exciting* | **Stressful**  **Confused**  **Moody**  **Lonely**  **Boring**  **Sad**  **Depressing**  **Out of Control** | *Happy*  *Courageous*  *Feeling Loved*  **Moody**  *Stable*  *Creative*  *Joyful*  *Relaxed*  *Confident*  *In control*  *Satisfying*  *Exciting* |

*Note:* Self-aspect titles have been adapted slightly for the purpose of anonymity, though they remain faithful to the content. Negative words are in bold, and positive words are in italics.
